# The social evolution of COVID-19: pandemics as *total social facts*

**DOI:** 10.3389/fsoc.2024.1397826

**Published:** 2024-07-08

**Authors:** Juan José Labora González, Enrique Fernández-Vilas

**Affiliations:** ^1^Department of Political Science and Sociology, Faculty of Political and Social Sciences, University of Santiago de Compostela, Santiago de Compostela, Spain; ^2^Department of Applied Economics, Faculty of Economics and Business Administration, University of Granada, Granada, Spain

**Keywords:** total social fact, COVID-19, pandemic, sociology of health, risk, Marcel Mauss

## Abstract

The COVID-19 pandemic was an unprecedented global event in recent history. Beginning with an initial outbreak in Wuhan, China, in December 2019, the virus spread rapidly across the globe, causing millions of deaths and triggering an unprecedented health, economic, and social crisis. The initial response to the outbreak in many countries was the implementation of social distancing measures, including the closure of schools and businesses, the cancellation of mass events, and the banning of travel. These measures were aimed at reducing the virus' spread and preventing health systems from being overwhelmed by the numerous severe COVID-19 cases. However, these measures also had a devastating economic impact, especially on precarious workers and freelancers, as well as those who were unable to work from home. As the pandemic (also considered a *syndemic* or *synergistic epidemic*) dragged on, countries adopted more flexible approaches to dealing with the virus, adopting mitigation measures rather than social distancing measures. These included the use of masks, testing and contact tracing, and the opening of businesses and schools with the implementation of additional safety measures. This paper highlights the social consequences of the pandemic, ultimately arguing that it is a *total social fact* (from the French *fait social total*), based on Marcel Mauss' categorization, since it encompassed and impacted all facets of human life.

## 1 Introduction

The COVID-19 pandemic (2020–2023) was an unprecedented global event in our recent history. Beginning with the initial outbreak occurring in Wuhan, China, in December 2019 (World Health Organization, 2020), the virus rapidly spread across the globe, causing millions of deaths and creating an unprecedented health, economic, and social crisis unlike anything experienced over the past hundred years.

The virus is spread by transmission between individuals through close contact -respiratory droplets- and contact with contaminated surfaces. The illness is characterized by a range of symptoms, including fever, cough, shortness of breath, and fatigue, and it is especially dangerous for older people (Liu et al., 2023) and those with pre-existing medical conditions (National Center for Immunization and Respiratory Diseases, 2022). Over time, it was determined that complete recovery from COVID tends to occur within 3–4 weeks after infection (see Davis et al., 2021; Hayes et al., 2021; López-León et al., 2021). This led to its classification into three categories: *Acute COVID-19*; and *Subacute COVID-19; Persistent COVID-19* or *Long COVID*.

*Long COVID* is characterized by persistent symptoms such as chronic fatigue, dyspnea, and multiple transient or fluctuating symptoms that can change over the course of the infection. The following main symptoms have been mentioned in the scientific literature: paresthesia, tinnitus, sleep disorders, post-traumatic stress syndrome, chest pain, and chronic kidney disease (Pinzon et al., 2022).

However, recent studies have highlighted that individuals suffering from long COVID experience persistent symptoms, notably asthenia and brain fog, which severely impair their ability to perform routine activities. Asthenia, characterized by an overwhelming sense of fatigue that is not alleviated by rest, and brain fog, a cognitive dysfunction marked by memory problems, a lack of mental clarity, and an inability to focus, suggest a broader, systemic involvement of the condition. This evidence underscores the complexity of long COVID, illustrating not only its direct impact on patients' physical health but also its profound effect on their cognitive functions and overall quality of life. The persistence of these symptoms indicates the need for a greater understanding of long COVID's pathophysiology and for comprehensive care strategies to address both the physical and cognitive challenges faced by affected individuals (see Gallegos et al., 2022).

Similarly, long COVID may cause a wide variety of symptoms in its sufferers, resulting in the following: the impossibility of returning to their prior work activity, an intense fear and concern due to the uncertainty caused by the illness, and limitations preventing their participation in sports or other daily activities. It has been suggested that in these patients, there may be cognitive alterations as well as those affecting their mental health (Badinlou et al., 2022). Approximately 144 million people across the globe suffer from long COVID, making up 3.64% of all infected individuals (Hanson et al., 2022). Moreover, women have been found to be more predisposed to this illness, although men tend to be affected by the related cognitive deficits (Astin et al., 2023).

Regardless, the initial response by most countries to the outbreak was to implement social distancing measures, despite the lack of evidence that these measures were effective, including closing schools (Rajmil, 2020), shutting down businesses, canceling large group events, and prohibiting travel. The objective of these measures was to reduce the spread of the virus and to prevent healthcare systems from being overwhelmed by the numerous serious COVID-19 cases. However, these measures also had a devastating economic impact, especially amongst precarious workers and freelancers, and those unable to work from home.

As the pandemic dragged on over 3 years (2020–2023), countries adopted more flexible approaches to deal with the virus, implementing mitigation as opposed to social distancing measures. These measures included the widespread use of masks, the implementation of testing and contact tracing, and the reopening of businesses and schools while implementing additional safety measures (*vid*. e.g., Koetter et al., 2020; Vaughn et al., 2023).

Uncertainty, risk, and lack of knowledge all created an unprecedented crisis affecting the health, economic, and social scenarios, in which distinct types of inequalities overlapped. This work highlights the social consequences of the pandemic, conceptually arguing that it is a *total social fact*, in accordance with the categorization established by French anthropologist Marcel Mauss last century (1923–1924/2016), since COVID-19 encompasses and impacts all facets of human life.

## 2 Marcel Mauss: *social fact* and overlap

From a sociological perspective, the pandemic embodies the paradigm of the risk society, characterized by systemic danger and global interconnectivity. The individual experience of the pandemic is perceived as a latent threat, generating a wide range of emotional responses which are both negative (anxiety, fear, sadness, grief) and positive (resilience, hope, trust in governmental management). The lack of complete knowledge regarding the genomic attributes of the virus results in a state of uncertainty, not as a conscious acknowledgment of the unknown (“we know that we do not know”), but rather, as a profound existential uncertainty (“we do not know what we do not know”) (Demertzis and Eyerman, 2020).

The mediation of the pandemic by the media has created a synthetic experience of the crisis, influenced by dramatization, personalization, and fragmentation of media coverage. This mediation affects the public's perception of the crisis and its associated risks, increasing the reliance on informational guidance within a context of limited information and heightened personal relevance. Diversity in media access and representation heightens conflicts over the interpretation of events and the allocation of responsibilities, fostering the proliferation of conspiracy theories and increasing uncertainty.

Furthermore, the pandemic has had a traumatic impact on society, with millions of people mourning the sudden loss of loved ones, often under extreme circumstances. Restrictions on mourning rituals, the vulnerability of older populations, and the isolation resulting from social distancing measures have created a situation of collective trauma. Desolate metropolitan areas and extreme measures implemented to handle the deceased have highlighted the magnitude of the pandemic's psychosocial and emotional impact.

This implies the search for relationships and correlations between specific elements of a study area, interpreting each element, not in isolation, but in reference to a broader structure of the examined phenomenon. Mauss suggested that a total social fact is embodied in the individual experience, encompassing two perspectives: an individual story and the total social fact itself, which Lévi-Strauss (1987) described as “anthropology,” understood as a system of interpretation that simultaneously explains all modes of behavior: physical, physiological, psychic, and sociological. This relates society to the individual, the social to the mental, seeking a “total” approach. This approach not only considers individual elements (such as art and life) separately but also as part of a whole. Mauss's concept is especially relevant in studies in those fields that are not hermetic, heterogeneous, or constantly changing (Ostaszewska, 2021).

In *Maussian* terms, the COVID-19 pandemic is a total social fact. In other words, it brings together all of the elements affecting people, from the sociological to the historical. When discussing total social facts, we are referring to phenomena affecting all spheres of life: the political, social, economic, cultural, educational, and health spheres, among others. The term was introduced by French anthropologist Mauss (2016), disciple and nephew of sociologist Émile Durkheim, who strongly influenced his thinking. Mauss used the concept to describe how social phenomena such as rituals and ceremonies may be considered “total social facts” affecting all aspects of the life of individuals in a given society:

The total social fact does not presuppose a parts-and-whole image of society along the lines of anthropology's functionalism. As far as Mauss is concerned, social life should not be understood through functional associations in the realms of economy, law, politics, religion, and so on; it manifests itself at its most condensed in specific situations where various economic, legal, political, and religious relationships overlap. The greatest obstacle here is the suspicion that these relationships are merely projections of our social categories. That is to say, it raises the question, familiar to anthropology, of how to overcome cognitive differences between societies (Kasuga, 2010, p. 101).

According to Mauss, total social facts are complete phenomena consisting of multiple elements, including cultural practices, institutions, beliefs, values, and symbols. These elements are interconnected and mutually reinforce one another so that the total social fact is a powerful connection of variables influencing the lives of individuals and how they think and act. Therefore, Mauss' idea is considered a manifestation of the interconnection of various elements in society, and its focus on this concept is related to the search to understand how social facts are composed and transformed through partial connections. These social facts are integral to the structure of society, and they affect all of its parts. By applying this concept to diseases, and specifically to the COVID-19 pandemic, it becomes clear how a health crisis can transform into a phenomenon that transcends the boundaries of public health, impacting and being influenced by a wide range of social dimensions.

The COVID-19 pandemic has been framed within this category since it forms part of the complex social phenomena affecting all aspects of an individual's life in a specific society and consisting of multiple interconnected elements. Within the context of the COVID-19 pandemic, this *Maussian* perspective invites us to examine how this health crisis has impacted all spheres of social life, and not only public health. Thus, the COVID-19 pandemic serves as a contemporary example of a “total social fact” since it extends to all spheres of life and consists of multiple interconnected elements. This perspective permits the understanding of the complexity of the pandemic and how its effects are intertwined in society in a manner that extends beyond a mere health crisis. This should be considered when implementing virus control or prevention measures, given that in this case, a biological element is being transmitted across individuals whose social dimension is affected by processes that form and permeate social groups and their interactions.

Therefore, social conditions matter, and they influence health and wellbeing. In his study on suicide, Durkheim (1951) already highlighted the importance of the solid social networks established between individuals and groups. However, it would not be until the 20th century when authors such as Thomas and Znaniecki (1996), in their classic sociological biographical work, The Polish Peasant in Europe and America, would clarify this fact. In the cited work, Thomas and Znaniecki interpreted the migratory process of a poor European population deciding to embark for the United States. Chicago was the specific destination of part of this migrant group. As a result of this, Chicago, which in 1840 was home to 4,470 residents, by 1930 had a total of 3,375,329 (Bulmer, 1984). This converted this city into an authentic melting pot of Germans, Scandinavian, Irish, Italians, Polish, Jews, Czechs, Lithuanians, and Croatians. It is calculated that in 1900, half of the population of 1.7 million people in this city had been born outside of the United States. The population of people of color rose from 2% in 1910 and to 7% by 1930 (Bulmer, 1984). Miranda (2009) noted that the population of people of color was ~250,000 people and that of white North Americans was only 27.5% of the city's total population.

All of this was added to an already complex situation resulting from the mafia's presence in the city, high levels of political and police corruption, the crisis caused by the Crash of 29, rising crime rates, and the Great Chicago Fire of 1871, affecting ~800 hectares and 17,500 buildings [with almost one out of every three city residents (some 70,000 people) being left homeless]. Thus, a high level of conflict was created across Chicago.

Due to this situation, a series of studies were launched at the University of Chicago's Sociology Department, supported by data from the empirical investigations underway by a majority of female social workers, led by Jane Addams and Mary Ellen Richmond and organized mainly by the Settlements movement founded by Addams, based on the experience of Toynbee Hall, founded by Samuel and Henrietta Barnett in 1884 (Lengermann and Niebrugge, 2006; Deegan, 2014). Based on the data obtained, a model was created, allowing for the interpretation of the city's social space, proposed by Robert Burgess (Burgess, 1927; Burguess and Bogue, 1964). The model proposed the existence of a series of concentric circles which, based on the force of the social conflict, sometimes generated centrifugal forces and sometimes produced centripetal forces, conveying social changes. That is, according to the Chicago School, the main hypothesis explaining the city declares that its internal structure does not evolve as a result of direct planning, but rather, by means of competition, which changes areas through the ecological processes of invasion, succession, and segregation of new groups.

From here, the authors currently known as the Chicago School coined the concept of “social disorganization”. This concept would permit the understanding of diverse phenomena such as rising delinquency (Landesco, 1929; Shaw and McKay, 1931; Shaw, 1938; Sutherland, 1956; Shaw et al., 2012), the phenomena of poor nomad workers, the so-called hobos (Anderson, 2014), the city as a social space and its dynamics (Wirth, 1964; Whyte, 2015), the situation of the African American population (Frazier, 1957; Drake and Cayton, 1993), or suicide (Sohnle, 1928).

Therefore, a phenomenon such as migration, at this time was analyzed as a total social phenomenon affecting population values, crime and delinquency, existing social conflicts, the form of population settlement in distinct areas of Chicago, and so on. Interestingly, in this case, Shaw -who had contact with the charitable organizations attempting to intervene in and alleviate the aforementioned problems, at the Chicago Urban League, founded in 1920 to coordinate social action in Chicago, discussed the results of the studies, as well as the measures taken to implement recommended actions. This had positive consequences over the middle and long term. That is, an analysis of migration from a holistic and complex perspective, ultimately modified the city's reality.

## 3 ¿Syndemic or *total social fact*?

Returning our focus to the analysis of the pandemic as a *total social fact*, the deterioration may be influenced by social variables and not purely biological ones. One of these variables is material conditions (Abrams and Szefler, 2020). To this we may add the position in society, social cohesion, or participation in public life since all of these elements are closely related to stress, as studies on this matter have consistently shown (Wiley and Allen, 2020).

The comprehensive social phenomena identified by Marcel Mauss are fundamental to society's structure, influencing every facet thereof:

For years, our attention has been on the regime of contract law and on the system of economic prestations (*prestations*) amongst the various sections and subgroups that make up so -called primitive societies, and also those societies that we could define as archaic. There is an enormous collection of facts there. And they themselves are very complex. Everything mixes in, everything that constitutes the life that is strictly social of the societies that have preceded our own- as far back as those of protohistory. In these “total” social phenomena, as we propose to call them, are expressed all at once and at a stroke all sorts of institutions: religious, judicial, and ethical (*morale*) -these being political and familial at the same time; economic- and they presume particular forms of production and consumption, or rather of prestation and distribution; without forgetting the aesthetic phenomena which bring things into final form, and the morphological phenomena manifested by these institutions (Mauss, 2016, p. 57–58).

Mauss introduced the term “total social fact” to describe phenomena that, within a society, simultaneously involve various institutions, practices, and spheres of social life. These phenomena are not limited to a single dimension (such as economic, legal, or moral), but affect and are affected by multiple aspects of collective life. The idea is that certain events or practices have the capacity to influence the entirety of the social fabric, encompassing and expressing the complexity of human relationships, their interactions, and their structures.

A total social fact, according to Mauss, is characterized by its ability to affect various areas of social life (economic, legal, moral, religious, etc.), involve the community as a whole, generating a collective impact, and manifest through practices that encompass exchanges, rituals, and social norms, reflecting the interconnection of social spheres.

When applied to health issues, specifically, to the COVID-19 pandemic, it becomes evident how a health crisis may evolve into a phenomenon that surpasses the realm of public health, affecting and being affected by a broad spectrum of social dimensions.

In economic terms, the pandemic had a major global impact, disrupting supply chains, forcing business closures, and escalating unemployment rates, leading to the most substantial global economic crisis in over a century. These economic repercussions underscore the extent to which a health crisis can influence a society's production, consumption, and financial stability (International Bank for Reconstruction and Development, 2022).

Furthermore, governmental responses to the pandemic, including lockdowns, border closures, and mask mandates, reflect the encompassing nature of total social facts through legal regulations and political decisions. These actions impact individual and collective rights, with their acceptance or resistance revealing underlying tensions within a society's political and legal framework (Alteri et al., 2021).

Socially and culturally, the pandemic has reshaped norms and behaviors, affecting greetings, social interactions, and behavior in large-scale events. It has also spotlighted and intensified social, racial, class, and gender inequalities, demonstrating how socioeconomic and cultural factors can influence a populations' vulnerability to illness (Wassler and Talarico, 2021).

Psychologically speaking, the pandemic has heightened anxiety and depression (Mertens et al., 2020), acting as a catalyst that exacerbates pre-existing concerns and unveils new forms of psychosocial vulnerability. This period, marked by a global surge in mood and anxiety disorders, has exposed alarming disparities in the impact of such conditions, disproportionately affecting already vulnerable groups. Some have described COVID-19 as a “cultural trauma” (Demertzis and Eyerman, 2020).

Recent studies have documented an increased prevalence of psychiatric pathologies, highlighting the urgent need for targeted and differentiated intervention approaches (e.g., Holmes et al., 2020; Hossain et al., 2020; Pappa et al., 2020; Winkler et al., 2020). The global health crisis has also intensified the manifestation of other disorders, such as Eating Disorders (ED) and Attention Deficit Hyperactivity Disorder (ADHD), reflecting the complexity and diversity of mental health responses to the pandemic (Jaguga and Kwobah, 2020; Miu et al., 2020; Tausch et al., 2022).

Religious practices and gatherings have played a dual role in the context of the COVID-19 pandemic, acting both as potential vectors for virus transmission and unique platforms for mobilizing and educating communities, especially minorities, on disease prevention and control measures. On the one hand, large gatherings and rituals involving physical congregations have been identified as contagion hotspots, highlighting the need to adapt religious practices to comply with public health guidelines. On the other hand, religious institutions have proven to be key actors in promoting health messages, leveraging their networks and outreach capabilities to disseminate vital information about the virus, encourage vaccination, and support vulnerable communities. This duality underscores the importance of collaborating with religious leaders and organizations in public health strategies, recognizing their influence and ability to reach broad, often marginalized population segments, fostering a more inclusive and effective response to the pandemic (Sisti et al., 2023).

Moreover, the race to develop vaccines and treatments for COVID-19 underscores the role of science and technology in addressing total social facts. The spread of information and misinformation about the pandemic through the social media and other digital channels highlights the significance of technology in shaping public perception and crisis response (Cinelli et al., 2020).

Similarly, the SARS-CoV-2 pandemic has emphasized the central role of material science in providing essential tools and technologies for advancing research and developing antiviral treatments. Previous initiatives in the field of material science aimed at creating advanced imaging systems and microfluidic devices have facilitated the detailed and real-time study of viral structures and transmission mechanisms. The exploration of platforms based on innovative materials for effective virus detection and the optimal delivery of antiviral drugs and vaccines has been specifically highlighted. The pivotal role of material science in producing personal protective equipment and developing simple, accurate, and cost-effective viral diagnostic devices underscores its undeniable importance in the global response (Tang et al., 2020).

Furthermore, the pandemic has exacerbated tensions between countries such as the United States and China over issues such as 5G technology, vaccine development, and disputes over the pandemic's origin (Demertzis and Eyerman, 2020).

The pandemic has radically transformed the educational landscape, requiring the adoption of distance learning and online education on an unprecedented scale, with significant disparities between countries. This shift has had profound implications for students, teachers, and parents, affecting not only how education is delivered but also its equity and accessibility. The digital divide has emerged as a critical issue, revealing how inequalities in access to technology and educational resources can exacerbate socioeconomic disparities (Kutsar and Kurvet-Käosaar, 2021). This aspect of the pandemic illustrates how a total social fact impacts the structure and functioning of the educational system, highlighting the intersections between technology, society, and the economy.

Regarding public health management, the COVID-19 pandemic has posed an unprecedented challenge to global public health systems, revealing structural vulnerabilities and response capacities to international health emergencies. Since its onset, this health crisis has placed extraordinary pressure on healthcare services, demanding the reallocation of resources to acute case management, the implementation of infection prevention strategies, and rapid research advances in order to better understand the pathogenesis, transmission, and treatment of SARS-CoV-2. It has also highlighted the importance of social determinants of health, including socioeconomic inequality and access to healthcare, on the spread and impact of the disease, underscoring the need to integrate public health approaches that promote health equity and justice, recognizing that public health dynamics result from a complex interplay of structural forces, including economic policies, socioeconomic development frameworks, social norms, and governance systems (Tulchinsky et al., 2023). The variability of these determinants across different social strata not only clarifies the heterogeneity observed in health indicators but also highlights the spectrum of existing inequalities and inequities (Nettleton, 2021; World Health Organization, 2024).

The response to the pandemic has demanded unprecedented levels of international collaboration in public health, involving multilateral organizations, national governments, the private sector, and civil society to coordinate efforts to contain the virus, develop and distribute vaccines, and implement non-pharmaceutical interventions (such as social distancing and mask-wearing). This situation has reinforced the relevance of concepts such as epidemiological surveillance, global health, and emergency preparedness, prompting a reassessment of public health systems worldwide. The pandemic has served as a catalyst for innovation in areas such as telemedicine, health data platforms, and risk communication, highlighting the importance of adaptability and resilience in health systems in the face of global health crises. Numerous studies have focused on these issues since the onset of the pandemic (e.g., Bambra et al., 2020; Greenaway et al., 2020; Krouse, 2020).

Finally, the COVID-19 crisis has raised significant ethical and moral challenges in public health, from the rationing of scarce medical resources to the implementation of vaccination policies and the management of data privacy in contact tracing. These ethical dilemmas reflect how the pandemic has forced societies to weigh individual rights against the common good, and how decisions in this realm may affect public trust in institutions and science (see Bellazzi and Boyneburgk, 2020; Robert et al., 2020; Wu and Kong, 2023). The way that different cultures and political systems have addressed these challenges serves to highlight the variability of ethical and moral norms across societies, underscoring the globally interconnected yet locally diverse nature of total social facts.

All of this reveals why the COVID-19 pandemic has had a disproportionate impact on low-income individuals and precarious workers (Santa-Ramírez et al., 2022), radicalized communities (Irizar et al., 2023), the elderly (Lithander et al., 2020), the disabled (Sohn et al., 2022), those living in rural, isolated areas or areas with poor Internet connection (Ferri et al., 2020), immigrants, refugees, asylum seekers and the displaced persons (Tazreiter and Metcalfe, 2021; Hitch et al., 2023), freelancers, informal economy workers (International Labour Organization, 2020), women (Wu and Qian, 2022) and children (Imran et al., 2020), the diagnosed mentally ill (Catalán et al., 2023), or the homeless (Abrams and Szefler, 2020; Sapey and Di Iorio, 2023), as well as other groups.

These inequalities have manifested in distinct aspects such as health, employment, education, housing, food security, access to public services, gender violence, or social exclusion. For this reason, in some sectors, there was talk of a *syndemic*, an idea proposed years earlier by Singer (2009), to refer to this relationship between factors. After years of affirming the integrity of the human being and the unsatisfactory bio-psycho-social models, the proposal of anthropologist Merrill Singer, presented in the mid-1990s and united in a 2009 book, demands special analysis extending beyond its specific context.

Upon introducing the *syndemics* concept, Singer suggested an interaction between causal factors, social processes, and pathological states that leads to a complex diversity of declarations of illnesses. If illnesses exist, their presentation is characterized by their diversity and variety. The same occurs with their consequences. Therefore, the term *syndemic* may be understood as the combination of “synergy” and “epidemic”, but it is preferable to consider the prefix “syn” as an indication of the fusion between conceptual horizons. This is precisely what is required, since a hasty search may obscure the need for understanding what is taking place and giving it an appropriate name (Lolas Stepke, 2020).

This idea of *syndemics*, in terms of public health, was visible with the COVID-19 pandemic since it highlighted the inequalities in access to health services and medical care. As previously mentioned, low-income and socially excluded communities were especially hard hit, due to their greater vulnerability to the disease and difficulties in accessing health services. In addition, the pandemic increased the workload in care services across the globe, leading to understaffing and overload in hospitals. Therefore, it is worth highlighting the ecological theories that emphasize the importance of taking early preventive measures to reduce exposure to health risk factors and prevent the accumulation of long-term damage. We should also note the importance of taking a holistic approach when addressing risk factors, considering the interaction of multiple factors and the distinct ways that they can affect health over time (causal accumulation). In the case of the COVID-19 pandemic, the infection has aggravated already existing risk factors, both psychosocial and biomedical.

When examining the COVID-19 pandemic through Marcel Mauss's lens of the total social fact, it becomes clear that this event is not merely a health crisis but is also a phenomenon that involves and impacts all dimensions of society. This perspective aids in the understanding of the complexity of responses to the pandemic and the interconnection of various aspects of social, economic, political, and cultural life.

The core difference between the concepts lies in their focus and application. While the total social fact refers to a broad phenomenon that encompasses multiple dimensions of social life, *syndemics* specifically focus on the interaction of diseases within a social context and the implications of these interactions on public health. Both concepts share the understanding that phenomena cannot be fully understood or addressed in isolation; they recognize the importance of considering the interaction between different factors (whether diseases, social or economic conditions, etc.) and their combined impact on society or population health.

According to Singer's idea of *syndemics*, considering these additional aspects increases our understanding of how the COVID-19 pandemic serves as a paradigmatic example of Marcel Mauss's total social fact concept. The crisis has impacted every facet of human life, illustrating how public health events can become catalysts for change and reflection across multiple societal domains. This holistic view permits an appreciation of the complexity of human responses to global crises and the importance of multidisciplinary approaches to tackle challenges that are, in essence, all-encompassing in their scope and impact.

## 4 Conclusion

The COVID-19 pandemic was an unprecedented global phenomenon, affecting roughly half of the world's population with three billion people being subjected to lockdowns during the initial months. The spread of the virus was facilitated by the globalization of transportation and international trade, leading to transmission at an unprecedented speed across all continents. This situation wreaked havoc on most national economies and global financial markets, while also causing political conflicts, ethical dilemmas, social segregation, and an increased prevalence of mental disorders. More than 4 years after the onset of the pandemic, over 650 million people have been infected and more than 6.8 million have died from the infection, as reflected in data from Johns Hopkins University (2023), based on an interactive map created to monitor real-time disease data. The globalized society led to this intense level of contagion. Simultaneously, and thanks to these advances, according to data from Johns Hopkins, more than 13 billion people have been vaccinated around the world.

Transmission of the SARS-CoV-2 virus, mainly through close contact and contaminated surfaces, has given rise to major challenges due to its ability to cause symptoms ranging from mild to severe, being especially dangerous for older people and those with pre-existing medical conditions. The classification of COVID-19 into the acute, subacute, and persistent (long COVID) forms has revealed a diversity of manifestations of the disease, with long COVID standing out due to its persistent and varied symptoms that significantly affect patient quality of life.

The impact of the pandemic goes beyond physical health, also deeply affecting mental health, with a notable increase in diagnoses of anxiety, depression, eating disorders, and other mental disorders, as well as a concerning increase in suicide rates. The pandemic has exacerbated pre-existing inequalities and has had disproportionate effects on vulnerable groups, highlighting socioeconomic, access to health, gender, and educational inequalities.

Initial response measures, such as social distancing and the closure of schools and businesses, although necessary to control the spread of the virus, have had devastating economic impacts, especially on precarious and self-employed workers. Over time, mitigation approaches such as mask-wearing, testing and contact tracing, and vaccination became key strategies. However, the pandemic has given rise to a far-reaching health, economic, and social crisis, exacerbating inequalities and highlighting the importance of social and cultural analysis in managing events of this magnitude.

The pandemic has also led to technological advances and transformed social relations and how individuals relate with one another, leading to the so-called “new normal”. This includes a new conception of urban space, with restrictions on mobility and the adoption of surveillance technologies to control the spread of the virus.

Finally, the paper highlights the relevance of health sociology and the sociology of risk within the context of the pandemic, noting how COVID-19 has impacted all facets of human life, becoming a “total social fact” in *Maussian* terms. This integral approach highlights the importance of considering the effects of the pandemic in a holistic manner, considering both the biomedical impacts as well as the psycho-social and socio-economic ones. The adoption of this type of analysis, which extends beyond concepts such as *syndemics*, permits a thorough study of complex phenomena such as the COVID-19 pandemic. This, in turn, eliminates the temptation to reduce health phenomena to their biomedical dimensions.

Furthermore, understanding the social dimensions of the processes and phenomena affecting health also permits the adoption of more effective prevention and intervention measures. These diseases are transmitted through interactions between people, and they are affected by the will of individuals to get vaccinated. Therefore, it is essential to determine how to communicate information and measures to ensure their understanding by the population. Otherwise, conspiracy theories will arise, as well as fake news and negationist movements, suggesting that an attempt is underway to limit personal freedom and promote social control by distinct governments through social engineering.

So once again, we must vindicate the role of the social sciences in general and health sociology, specifically, in the understanding of health-related phenomena, since they are decisively influenced by the individual's social dimension; this potentially converts this type of phenomena into a total social phenomenon in which the individual's social condition may even engulf the biological dimension. Paradoxically, however, governments and authorities tend to overlook this, continuing to apply strictly biological measures to social phenomena. Thus, from this pandemic, we should learn the lesson of implementing complex, holistic analyses, and having a multidisciplinary—if not interdisciplinary—nature, to improve the results of measures adopted in complex situations affecting people who have both bodies and minds. People clearly have social dimensions; therefore, ignoring this dimension may lead to mental health issues such as isolation, exclusion, or new vulnerabilities and forms of discrimination in certain sectors of the population.

## Author contributions

JL: Conceptualization, Investigation, Methodology, Supervision, Writing – original draft, Writing – review & editing. EF-V: Conceptualization, Formal analysis, Investigation, Writing – original draft, Writing – review & editing.
